# A Personal Appeal

**Published:** 1877-08

**Authors:** 


### A PERSONAL APPEAL

To able writers and eminent publishers to supply families with a monthly reading differing from that of any other monthly publication known at this time——— a periodical not claiming to be religious, yet to be ALWAYS on the side of sound morals and an evangelical Christianity. Fictitious reading to be almost entirely, if not altogether excluded.

There is one chief reason why such a Monthly should be published, and should be patronized by all good men and true:

The weekly and monthly publications which have by far the widest circulation in the country, and which are not professedly religious, largely abound in fictitious reading, and are written for, to a too great extent, by men whose principles are levelling and infidel—an infidelity not far from a practical atheism. Some of these are men of mind, of genius, of science, and of a high culture, are splendid writers, and too often use their power and opportunity in darting into the mind of their readers arrows barbed with a rankling poison, which, when once carried home to the heart of the young, at an opportune moment, remains there ever after, to fester, and worry, and unsettle, if not to destroy all religious belief. It is simply hoped that some families of the many who cannot afford two Monthlies, but will take one, may decide to take this in preference, as being at least safe, even if it does not give as much reading matter. Parents will not fail to observe that the generality of the Weeklies and Monthlies, not claiming to be religious, contain so much reading, and that very largely fictitious or utterly frivolous, that it requires nearly all the "spare time," so called, to read them; and more, time is too often spent upon them which ought not to be spared from the necessary duties and avocations of life. It is truly believed, therefore, that those parents who would not for a world that a child of theirs should grow up to be an "infidel," will consult the present and future welfare of themselves and their offspring, by taking such a MONTHLY, in preference to such publications as those above referred to.

